# Heat Shock Protein 60 Overexpression Is Associated with the Progression and Prognosis in Gastric Cancer

**DOI:** 10.1371/journal.pone.0107507

**Published:** 2014-09-10

**Authors:** Xiao-shan Li, Qing Xu, Xiang-yang Fu, Wei-sheng Luo

**Affiliations:** 1 Department of Gastroenterology, Affiliated Hospital of Guilin Medical University, Guilin, Guangxi Zhuang Autonomous Region, China; 2 Guilin Medical University, Guilin, Guangxi Zhuang Autonomous Region, China; 3 Department of Spleen and Stomach Diseases, The First Affiliated Hospital of Guangxi University of Chinese Medicine, Nanning, Guangxi Zhuang Autonomous Region, China; University of Palermo, Italy

## Abstract

**Background:**

Heat shock protein 60 (HSP60) is a chaperonin with essential functions for cell physiology and survival, and its expression correlates with prognosis in a number of malignancies. The aim of this study is to determine the relationship of HSP60 status with clinicopathological parameters and prognosis in gastric cancer.

**Methods:**

The levels of HSP60 and matrix metallopeptidase 9 (MMP-9) antigen was evaluated by immunohistochemistry in 223 gastric carcinoma samples. The association between HSP60 and MMP-9, clinicopathological parameters, and prognosis of gastric cancer was examined.

**Results:**

The level of HSP60 protein was significantly associated with depth invasion, lymph node metastasis and stage of disease (all *P*<0.05). Both univariate and multivariate analyses revealed that HSP60 was an independent prognostic factor for both overall survival (OS) and recurrence-free survival (RFS) (both *P*<0.05). Furthermore, HSP60 overexpression was associated with a poor prognosis in patients with advanced gastric cancer in different risk groups. Moreover, HSP60 was significantly correlated with MMP-9 among 223 gastric cancer tissues (*P*<0.001). Patients who had HSP60 overexpression, in which tumor cells displayed high invasiveness, had poor OS and shorter RFS.

**Conclusion:**

HSP60 plays an important role on tumor aggressiveness and prognosis, and may act as a promising target for prognostic prediction.

## Introduction

Gastric cancer is one of the leading causes of cancer-related death worldwide due to its frequency, poor prognosis and limited treatment options [1]. Complete resection of the tumor and adjacent lymph nodes is the only effective curative treatment [2]. Unfortunately, after a complete resection, the 5-year survival rate remains low [3]. Several studies have shown that various genetic and epigenetic alterations are involved in the course of carcinogenesis and progression of gastric cancer [4]–[6]. However, the molecular mechanism involved in the development of gastric cancer remains unclear.

Heat shock proteins (HSPs) are a set of evolutionarily conserved proteins. HSPs have been classified into various subfamilies based on their molecular weight [7] or systematic gene symbols [8]. Broadly, in mammals, four major families of HSPs are recognized: HSP90 (HSP90α, HSP90β, GRP94), HSP70 (HSP70, HSC70, mHSP70, GRP78), HSP60 and small HSPs (HSP27, αB-crystallin, HSP22). Accumulation of misfolded protein in stressed cells, triggers expressions of HSPs, which prevent protein aggregation and facilitate refolding or elimination of misfolded proteins in their capacities as chaperones [9]. Recently, it has been reported that HSPs are significantly associated with cancers [10].

HSP60 (also called Cpn60) is mostly contained within the mitochondrial matrix, although it has also been detected at extra-mitochondrial sites [11]. HSP60 assists the folding of mitochondrial proteins and facilitates proteolytic degradation of misfolded or denatured proteins in an ATP-dependent manner [12]. Recently, its role and applications in human cancer development and management are currently actively investigated and the results are very encouraging [13]. HSP60 seems to have potential in the areas of diagnosis, prognosis, and prevention and treatment of various human cancers, including oesophageal squamous cell carcinoma [14], bronchial carcinogenesis [15], colorectal cancer [16] and glioblastoma [17]. However, the role of HSP60 on the prognosis of patients with gastric cancer remains unclear.

In the present study, we assessed the level of HSP60 in gastric cancer tissues by immunohistochemistry. Correlations of HSP60 status with clinicopathological parameters and survival of gastric cancer patients was then analyzed. In addition, it has been reported that MMP-9 plays an important role in gastric cancer recurrence and prognosis [18]. Therefore, we also investigated the relationship of HSP60 and MMP-9 protein in gastric cancer.

## Patients and Methods

### Patients and Specimens

The study was approved by the Institutional Review Board and Human Ethics Committee of Affiliated Hospital of Guilin Medical University. Written consent for using the samples for research purposes was obtained from all patients prior to surgery.

Gastric carcinoma tissues were obtained from gastrectomy specimens of 223 patients from the department of general surgery, the Affiliated Hospital of Guilin Medical University (Guilin, China). All the operations were performed between January 2005 and December 2008. The eligibility criteria of the current study were as follows: (1) a pathologic examination confirming the presence of gastric cancer and experienced radical surgery, (2) complete basic clinical data, (3) the absence of any prior treatment for cancer, and (4) no serious complications or other malignant disease. There were 144 males and 79 females (median age, 58.0 years; range, 22–82 years). Relevant clinical pathologic features (Table 1) were all obtained from the patients’ files. Tumor stage was classified according to the 7th Union International Cancer Control (UICC) TNM staging system [19].

**Table 1:** Clinicopathologic Correlation of HSP60 Status in 223 Gastric Cancer.

Characteristics	No. of patients	Level of HSP60 (%)		*P*-value
		Negative	Positive	
Gender				
Male	144	56 (38.9%)	88 (61.1%)	
Female	79	37 (46.8%)	42 (53.2%)	0.250
Age (years)				
≤60	140	63 (45.0%)	77 (55.0%)	
>60	83	30 (36.1%)	53 (63.9%)	0.195
Size (cm)				
≤5.0	146	67 (45.9%)	79 (54.1%)	
>5.0	77	26 (33.8%)	51 (63.2%)	0.081
Tumor site				
Upper	88	32 (36.4%)	56 (63.6%)	
Middle/Lower	135	61 (45.2%)	74 (54.8%)	0.192
Differentiation				
Well/Moderate	100	42 (42.0%)	58 (58.0%)	
Poor	123	51 (41.5%)	72 (58.5%)	0.936
Depth of invasion				
T1/T2	88	44 (50.0%)	44 (50.0%)	
T3/T4	135	49 (36.3%)	86 (63.7%)	**0.043**
Lymph node metastasis				
Negative	65	35 (53.8%)	30 (46.2%)	
Positive	158	58 (36.7%)	100 (63.3%)	**0.018**
Stages				
I/II	112	54 (48.2%)	58 (51.8%)	
III	111	39 (35.1%)	72 (64.9%)	**0.048**

### Immunohistochemistry staining

A total of 223 gastric carcinoma samples were used in the immunohistochemistry (IHC) analysis. According to protocol [20] for IHC on paraffin-embedded tissue sections, paraffin-embedded blocks were sectioned at about 4 µm thickness. Slides were baked at 60°C for 2 h, deparaffinized with xylene and rehydrated using an alcohol gradient (100% alcohol, 95% alcohol, 80% alcohol, and 70% alcohol). The tissue slides were then treated with 3% hydrogen peroxide in methanol for 30 min to quench endogenous peroxidase activity, and the antigens were retrieved in 0.01 M sodium citrate buffer (pH 6.0) using a microwave oven. After 30 min of preincubation in 10% normal goat serum to prevent nonspecific staining, the samples were incubated overnight using a primary antibody, either anti-HSP60 (Abcam, #ab31115, UK, dilution 1∶200) or anti-MMP-9 (Abcam, #ab38898, UK, dilution 1∶200), in a humidified container at 4°C. The tissue slides were treated with a non-biotin horseradish-peroxidase detection system according to the manufacturer’s instructions (Gene Tech). The IHC results were evaluated by two independent investigators blinded to the patients’ identity and clinical status. In discrepant cases, a pathologist reviewed the cases, and a consensus was reached.

**Figure 1:**
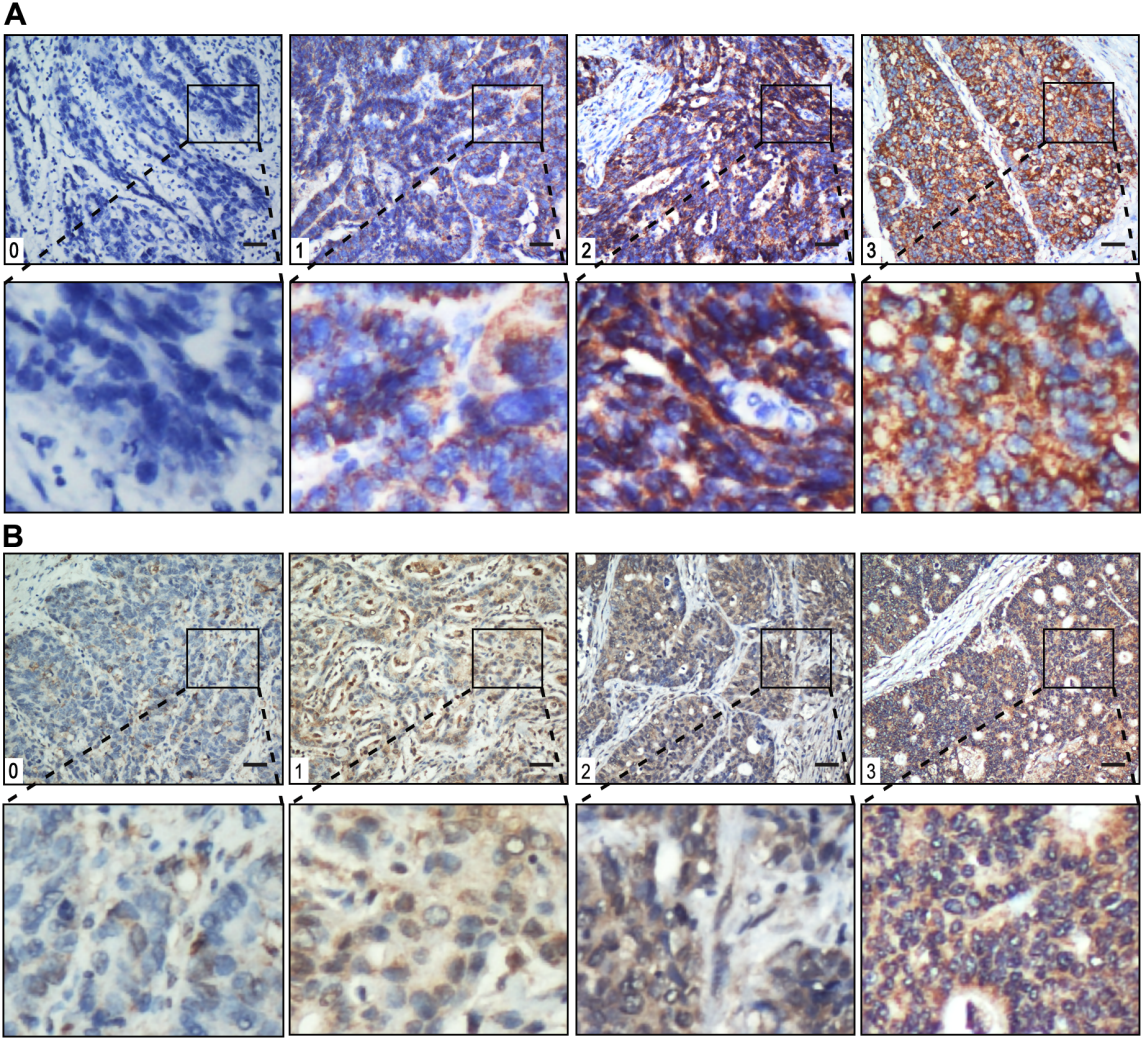
Gastric cancer tissue illustrating the range of intensities of HSP60 (A) and MMP-9 (B) immunostaining from 0 to 3. The lower panels in (A) and (B) represent magnified pictures of boxed area in the corresponding upper panels. The scale bar represents 100 µm.

HSP60 and MMP-9 staining intensities were rated on a scale of 0–3 according to the percentage of positive tumor (0, <5% positive cells; 1, 5–10%; 2, 11–50%; or 3, >50%). The level is very low for 0, low for 1, moderate for 2 and high for 3 (Fig. 1). The levels of HSP60 and MMP-9 were classified as negative for scores ≤1 and positive for scores ≥2.

### Follow-up

The follow-up duration was defined as the interval between the date of operation and the date of death or last follow-up. The study was censored on 30 September 2013. The median follow-up period was 27.0 months (range, 4–82 months) in 223 patients. All the patients were followed up every 1–3 months in the first year and every 3–6 months thereafter. Recurrence were confirmed by tumor markers levels including CEA, AFP, CA199, CA125 and CA724, B-type ultrasonic inspection every 3 moths, and computed tomography (CT) or magnetic resonance imaging (MRI) every 6 months after gastrectomy. The main causes of death were gastric cancer recurrence. Overall survival (OS) was calculated from the date of surgery to the date of death or last follow-up. Recurrence-free survival (RFS) was defined as from the date of surgery until the date of relapse or from the period of resection to the date of the last observation taken.

### Statistical analysis

All statistical analyses were performed using the SPSS software (version 16.0; Chicago, IL, USA). Interdependence between HSP60 and clinical data was calculated using the chi-square test, and displayed in cross-tables. Correlation of HSP60 with MMP-9 was calculated by Pearson χ2 test. Survival curves were plotted using the Kaplan-Meier method and analyzed using the log-rank test. All reported *P* values were two-sided and *P*<0.05 was considered statistically significant.

## Results

### The association of HSP60 with clinicopathological variables

To elucidate the biological significance of HSP60 in gastric cancer, we examined the immunohistochemical level of HSP60 in gastric cancer tissues (Fig. 1). HSP60 staining mainly located in cytoplasm of tumor cells. The positive rate of HSP60 was 58.3% (130/223) in gastric cancer samples.

According to the results of immunohistochemistry, we correlated HSP60 status in 223 gastric cancer specimens with eight other widely recognized clinicopathologic parameters (Table 1). Our analyses showed that the level of HSP60 in gastric cancer was significantly correlated with depth of invasion (*P* = 0.043), lymph node metastasis (*P* = 0.018) and stage of disease (*P* = 0.048), but was not associated with gender, age, tumor size, tumor site and grade of differentiation (*P*>0.05) (Table 1). Notably, the correlation of HSP60 status with prominent serosal invasion and lymph node metastasis positivity suggested a potential role of HSP60 in increased invasion and metastasis of gastric cancer.

### Effect of tumor HSP60 protein level on prognosis

To further determine the effects of HSP60 on the OS and RFS, we first performed univariate analysis of traditional clinicopathologic variables for prognosis. The results of the univariate analysis are shown in Table 2. Overexpression of HSP60 (*P* = 0.001), larger tumor size (*P*<0.001), tumor site (*P* = 0.014), prominent serosal invasion (*P*<0.001) and lymph node metastasis (*P*<0.001) were significantly associated with the poor OS rate of gastric cancer patients. In addition, Kaplan-Meier analysis demonstrated that HSP60 overexpression (*P* = 0.002), larger tumor size (*P*<0.001), tumor site (*P* = 0.008), prominent serosal invasion (*P*<0.001) and lymph node metastasis (*P*<0.001) were negative prognostic factors for RFS in gastric cancer patients (Table 2). Furthermore, to evaluate the independent impact of HSP60 overexpression on OS and RFS, a multivariate Cox regression model adjusted for tumor size, tumor site, depth of invasion, lymph node metastasis and HSP60 was performed. Our results showed that HSP60 status was a poor independent prognostic factor for OS in gastric cancer patients (hazard ratio, 1.594; 95% CI, 1.114–2.280). In addition, positive HSP60 expression patients were almost 1.5 times more likely to suffer from relapse than those with negative HSP60 expression (hazard ratio, 1.460; 95% CI, 1.024–2.081). Tumor size, depth of invasion and lymph node metastasis all had independent prognostic value in the multivariate analysis (Table 3).

**Table 2:** Predictive Variables for Overall Survival and Recurrence-Free Survival of 223 Patients with Gastric Cancer.

Variables	No. of patients	OS rate (%)		*P*-value	RFS rate (%)		*P*-value
		3 y	5 y		3 y	5 y	
Gender							
Male	144	46.3	37.9		41.0	33.7	
Female	79	55.2	38.9	0.300	44.4	38.5	0.306
Age (years)							
≤60	140	50.5	41.1		45.4	40.7	
>60	83	47.6	33.7	0.330	36.8	27.5	0.271
Size (cm)							
≤5.0	146	56.5	47.2		50.7	44.3	
>5.0	77	36.2	22.3	**<0.001**	26.4	19.3	**<0.001**
Tumor site							
Upper	88	42.9	29.9		32.7	20.7	
Middle/Lower	135	53.7	43.6	**0.014**	48.3	44.0	**0.008**
Differentiation							
Well/Moderate	100	48.6	39.5		39.8	32.5	
Poor	123	50.0	37.1	0.748	44.1	37.9	0.726
Depth of invasion							
T1/T2	88	75.0	67.8		72.9	64.3	
T3/T4	135	32.0	17.5	**<0.001**	20.8	14.9	**<0.001**
Lymph node metastasis							
Negative	65	85.3	77.1		82.3	72.7	
Positive	158	34.2	21.8	**<0.001**	24.9	19.2	**<0.001**
HSP60 protein status							
Negative	93	64.1	48.9		53.7	43.5	
Positive	130	38.6	30.2	**0.001**	33.7	29.3	**0.002**

**Table 3:** Multivariate Cox Regression Analysis for Overall Survival and Recurrence-Free Survival in Patients with Gastric Cancer.

Variable	OS (n = 223)		RFS (n = 223)	
	Hazard Ratios (95% CI)	*P*-value^a^	Hazard ratios (95% CI)	*P*-value^a^
Tumor size (cm)(≤5.0 *versus*>5)	1.450 (1.021∼2.060)	**0.038**	1.462 (1.033∼2.069)	**0.032**
Tumor site(upper *versus* middle/lower)	1.017 (0.712∼1.453)	0.927	0.937 (0.660∼1.331)	0.718
Depth of invasion(T1/T2 *versus* T3/T4)	2.500 (1.498∼4.175)	**<0.001**	2.363 (1.434∼3.892)	**0.001**
Lymph node metastasis(negative *versus* positive)	2.986 (1.568∼5.688)	**0.001**	3.238 (1.711∼6.126)	**<0.001**
HSP60(negative *versus* positive)	1.594 (1.114∼2.280)	**0.011**	1.460 (1.024∼2.081)	**0.036**

Abbreviations: HSP60, heat shock protein 60; CI, confidence interval.

a Cox proportional hazard model regression. Bold values are statistically significant (*P*<0.05).

**Figure 2:**
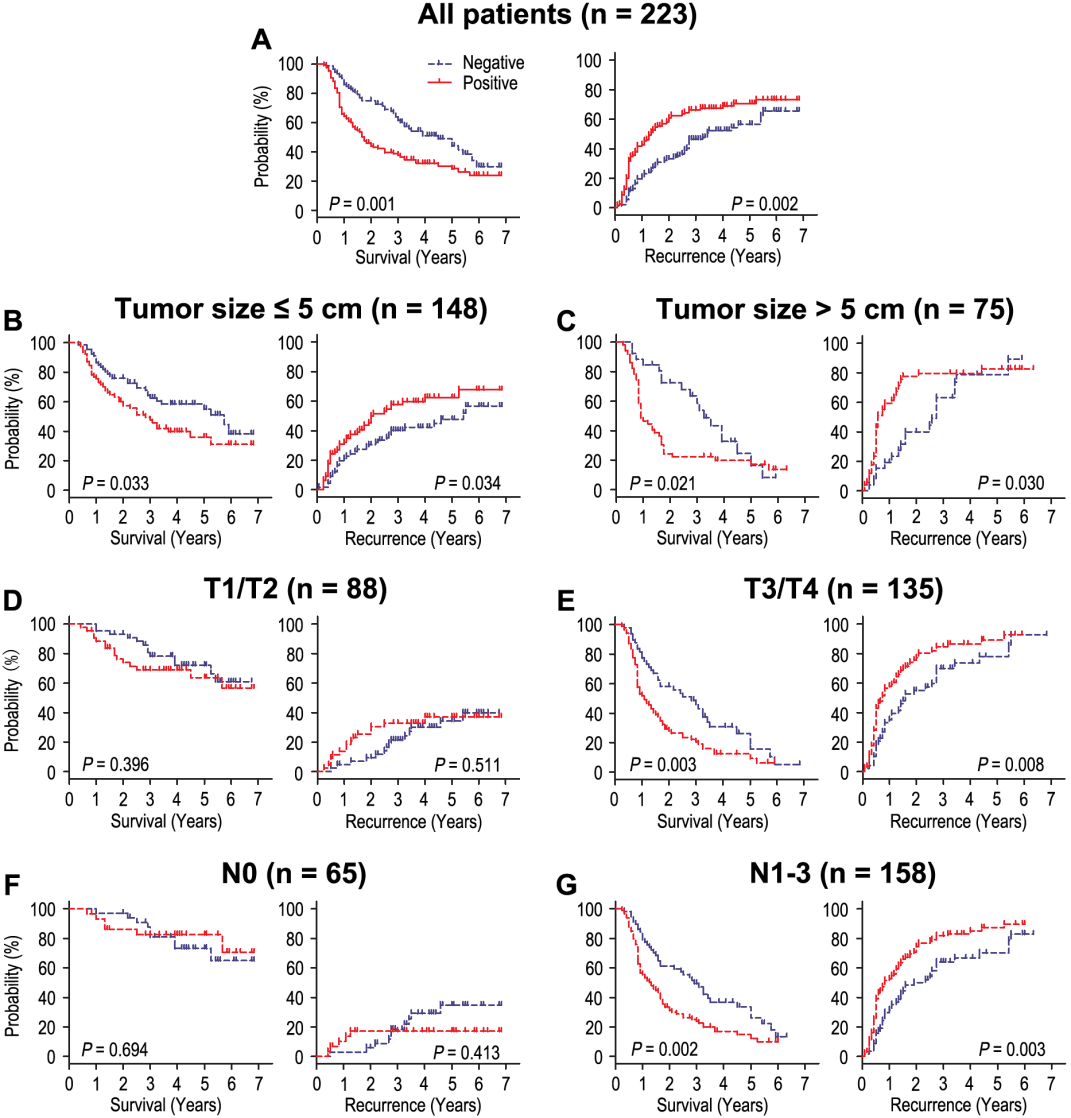
Overall survival and recurrence-free survival are shown for patients with gastric cancer. All patients were stratified according to tumor size, depth of invasion and lymph node metastasis. Kaplan-Meier survival estimates and log-rank tests were used to analyze the prognostic significance of HSP60 in all patients (A) and each subgroup (B–G).

Survival analysis showed that OS and RFS were significant different among 223 patients according to the level of HSP60 (*P* = 0.001, *P* = 0.002) (Fig. 2A). The postoperative median OS and RFS were 26.0 months and 20.0 months, respectively. The postoperative median OS times in HSP60-positive (n = 130) and HSP60-negative (n = 93) gastric cancer patients subgroup were 17.5 months and 36.0 months, and the median of RFS times were 14.0 months and 33.0 months. In addition, the OS and RFS rates at 5 years were 30.2% and 29.3% for HSP60-positive patients compared with 48.9% and 43.5% for HSP60-negative patients, respectively (*P* = 0.001 and *P* = 0.002; Table 2).

To further evaluate the prognostic value of HSP60 in different subgroups, patients were stratified according to tumor size (Fig. 2B,C), depth of invasion (Fig. 2D,E) and lymph node metastasis (Fig. 2F,G). The level of HSP60 maintained its prognostic value in predicting shorter OS and RFS in the subgroups for tumor size. For the subgroups of T3/T4 and N1–3 patients, significant correlations were found between HSP60 status and OS (*P* = 0.003 and *P* = 0.002; respectively) and RFS (*P* = 0.008 and *P* = 0.003; respectively). HSP60 had no prognostic value regarding OS or RFS for patients with T1/T2 and N0 (all *P*>0.05). Therefore, it appears that HSP60 may serve as a powerful prognostic factor for patients with advanced gastric cancer in different risk groups.

### HSP60 overexpression predict poor prognosis independent of tumor invasiveness

**Figure 3:**
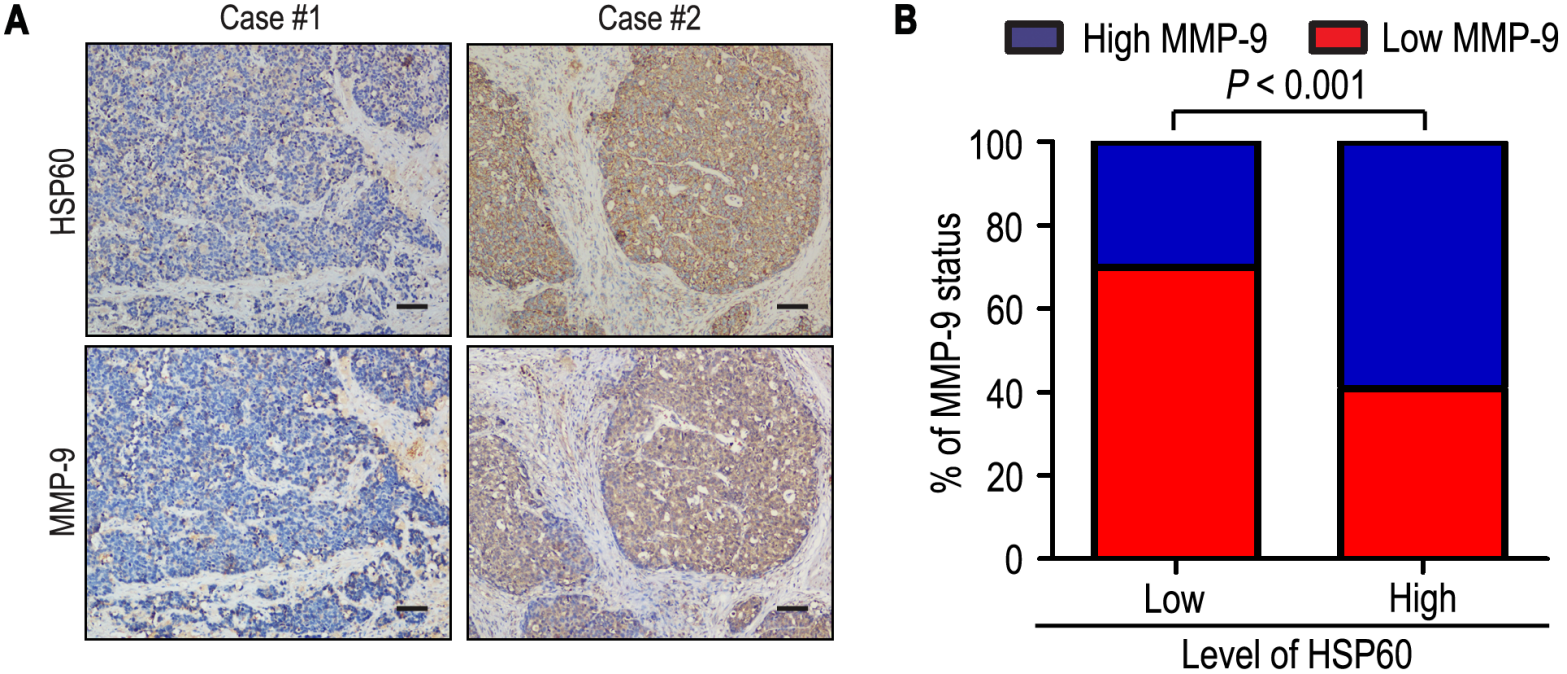
HSP60 and MMP-9 protein levels correlated in 223 gastric cancer tissues. (**A**, **B**) IHC staining for HSP60 and MMP-9 was performed in tumors from 223 gastric cancer patients. Representative examples of HSP60 and MMP-9 staining in serial sections from the same tumor samples are shown in (**A**), and percentages of samples displaying low or high level of HSP60 relative to MMP-9 level is shown in (**B**). The scale bar represents 200 µm.

To better understand the clinical significance of HSP60 on aggressiveness in gastric cancer, we investigated the relationship of HSP60 with depth invasion and lymph node metastasis in gastric cancer. The positive rates of HSP60 were 63.7% and 63.3% in the more prominent serosal invasion group (T3/T4) and more frequent lymph node involvement group (N1–3), while there were only 50.0% and 46.2% in T1/T2 and N0 (*P* = 0.043 and *P*<0.018, respectively) (Table 1). In addition, the level of HSP60 was significantly correlated with MMP-9 in 223 gastric carcinoma specimens. Of 93 patients with low level of HSP60, 65 patients (69.9%) had low level of MMP-9, while 77 of 130 patients (59.2%) with high level of HSP60 also had high level of MMP-9 (*P*<0.001) (Fig. 3).

**Figure 4:**
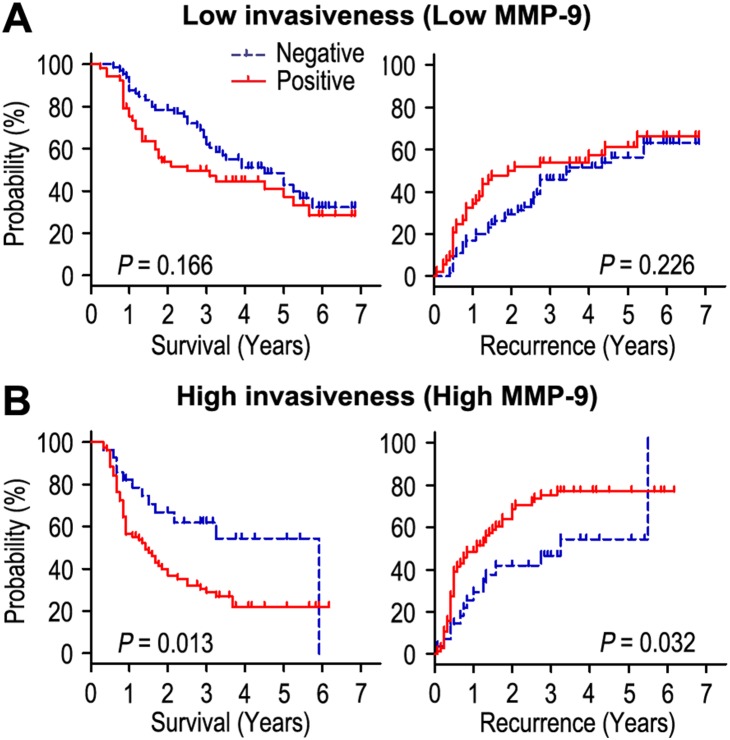
Overall survival and recurrence-free survival are shown for patients with high tumor invasiveness (A) and low tumor invasiveness (B). Kaplan-Meier survival estimates and log-rank tests were used to analyze the association between HSP60 status and overall survival or recurrence-free survival in patients with low invasiveness (low MMP-9; n = 118) or high invasiveness (high MMP-9; n = 105).

We further explored the influence of tumor invasiveness on the prognostic value of HSP60 in gastric cancer by using MMP-9 as an indicator for the invasive potential of individual tumor cells. All the patients were stratified into either a low invasiveness subgroup (low MMP-9; n = 118) or a high invasiveness subgroup (high MMP-9; n = 105) according to the level of MMP-9 index. Kaplan-Meier survival curves were then plotted to investigate the association between HSP60 status and survival (Fig. 4). In the high tumor invasiveness subgroup, HSP60 overexpression was associated with shorter OS (*P* = 0.013) and RFS (*P* = 0.032) compared with the OS and RFS in patients with low level of HSP60, while there was no prognostic value regarding OS or RFS in the low invasiveness subgroup.

## Discussion

In the present study, the level of HSP60 was investigated in 223 gastric carcinoma tissues by immunohistochemistry. We found that HSP60 status was significantly associated with depth invasion and lymph node metastasis. In addition, the Kaplan-Meier survival analysis revealed that the survival times (OS and RFS) of gastric cancer patients with HSP60 overexpression were significantly shorter than those with low level of HSP60. Furthermore, the multivariate Cox model analysis indicated that HSP60 status was an independent factor for both prognosis indexes (OS and RFS) in gastric cancer. This finding suggests that HSP60 plays an important role in tumor prognosis, and could be a potential prognostic factor of gastric cancer. Our results were consistent with previously reported results. In several investigations, it has been shown that the abnormal expression of HSP60 in cancer cells is associated with tumor progression. Tanaka et al. [21] reported that the positivity rate of H. pylori HSP60 was significantly higher in patients with diffuse-type gastric cancer than in H. pylori-positive patients without gastric cancer, which suggested that H. pylori HSP60 might be associated with gastric carcinogenesis, especially in the case of diffuse cancer. Faried et al. [14] compared the correlation of HSP60 status with clinicopathological parameters and prognosis in oesophageal squamous cell carcinoma and suggested that HSP60 correlated with patient’s prognosis in human oesophageal squamous cell carcinoma. In addition, Cappello et al. [22] evaluated the presence and expression of HSP60 and HSP10 in bowel carcinomas with lymph node metastasis and found that HSP60 and HSP10 overexpression was functionally related to tumoral progression in bowel carcinomas. However, Cappello et al. [23] compared the HSP60 and HSP10 expression in lung cancer with that of chronic obstructive pulmonary disease by immunohistochemistry and found the contradictory results that the loss of HSP60 and HSP10 immunopositivity is related to the development and progression of bronchial cancer in smokers with chronic obstructive pulmonary disease.

Clinical stage is the most important factor influencing the prognosis of gastric cancer patients. Several systems are available to classify gastric cancer. Among them, the Union International Cancer Control (UICC) TNM staging system is one of the most prevalent. Although the TNM system has successfully graded patients on their prognosis according to clinicopathological variables, it has reached its limit in providing critical information that may influence treatment strategy. It is difficult for gastrointestinal surgeons to predict exactly which individuals will experience relapse among advanced patients who have undergone curative treatment. To overcome the limitations of these traditional systems, many molecular markers have been investigated and shown to have potential predictive significance. However, to date, biomarkers that could stratify gastric cancer patients with curative excision in TNM stage III/IV are still substantially limited. In our stratified analysis, we found that HSP60 status had clear prognostic value for OS and RFS in T3/T4 and lymph node metastasis (N1–3) patients. These data imply that HSP60 might act as a predictive tool to identify patients with advanced gastric cancer at high risk of recurrence.

It has been known that degradation of extracellular matrix (ECM) was a signal for the beginning of invasion and metastasis, and MMPs are important molecules involved in ECM degradation during invasion and metastasis [24]. Chu et al. [18] reported that cancer MMP-9 was significantly correlated with depth of invasion and lymph node metastasis and MMP-9-positive gastric cancer patients had worse outcomes than those with MMP-9-negative tumors. Zhao et al. [25] found that MMP-9 targeted RNA interference was able to successfully suppress MMP-9 expression and inhibit cell growth and invasion of SGC7901 gastric cancer in vitro and in vivo. In addition, Lin et al. [26] reported that the heat shock protein 60 of H. pylori enhanced migration by gastric cancer cells and promoted tube formation, moreover, it could accelerate cancer development in the way of expressing the pro-inflammatory cytokines. It has been reported that HSP60 is proposed as target for tumor therapy [27]. Our results demonstrated that the level of HSP60 and MMP-9 were correlated with each other, indicating higher invasive and metastasizing activity in HSP60-positive cancer cells. In addition, HSP60 was highly expressed in depth of invasion, especially in T3 and T4 carcinomas. As far as lymph node status was concerned, the patients with lymph node metastasis tend to show elevated HSP60 expression. Furthermore, patients who had HSP60 overexpression, in which tumor cells displayed high invasiveness, had poor OS and shorter RFS. Collectively, HSP60 in gastric cancer promoting tumor aggressiveness suggests that HSP60 could be a feasible target in cancer therapy.

In summary, we demonstrated that HSP60 may play an important role in tumor invasion, metastasis and prognosis, and could work as a promising target for prognostic prediction in gastric cancer. Determination of HSP60 may help to identify high-risk gastric cancer patients and thus aid the selection of appropriate therapies. Further investigation is necessary to clarify the role of HSP60 in the development of gastric cancer.

## References

[1] Compare D, Rocco A, Nardone G (2010) Risk factors in gastric cancer. Eur Rev Med Pharmacol Sci 14: 302–308. 20496539

[2] Dikken JL, van Sandick JW, Maurits Swellengrebel HA, Lind PA, Putter H, et al (2011) Neo-adjuvant chemotherapy followed by surgery and chemotherapy or by surgery and chemoradiotherapy for patients with resectable gastric cancer (CRITICS). BMC Cancer 11: 329. 21810227 10.1186/1471-2407-11-329 PMC3175221

[3] Cunningham D, Allum WH, Stenning SP, Thompson JN, Van de Velde CJ, et al (2006) Perioperative chemotherapy versus surgery alone for resectable gastroesophageal cancer. N Engl J Med 355: 11–20. 16822992 10.1056/NEJMoa055531

[4] Nobili S, Bruno L, Landini I, Napoli C, Bechi P, et al (2011) Genomic and genetic alterations influence the progression of gastric cancer. World J Gastroenterol 17: 290–299. 21253387 10.3748/wjg.v17.i3.290 PMC3022288

[5] Yasui W, Sentani K, Sakamoto N, Anami K, Naito Y, et al (2011) Molecular pathology of gastric cancer: research and practice. Pathol Res Pract 207: 608–612. 22005013 10.1016/j.prp.2011.09.006

[6] Bornschein J, Rokkas T, Selgrad M, Malfertheiner P (2011) Gastric cancer: clinical aspects, epidemiology and molecular background. Helicobacter 16 Suppl 1 45–52. 21896085 10.1111/j.1523-5378.2011.00880.x

[7] Garrido C, Gurbuxani S, Ravagnan L, Kroemer G (2001) Heat shock proteins: endogenous modulators of apoptotic cell death. Biochem Biophys Res Commun 286: 433–442. 11511077 10.1006/bbrc.2001.5427

[8] Kampinga HH, Hageman J, Vos MJ, Kubota H, Tanguay RM, et al (2009) Guidelines for the nomenclature of the human heat shock proteins. Cell Stress Chaperones 14: 105–111. 18663603 10.1007/s12192-008-0068-7 PMC2673902

[9] Kim HJ, Hwang NR, Lee KJ (2007) Heat shock responses for understanding diseases of protein denaturation. Mol Cells 23: 123–131. 17464187

[10] Calderwood SK, Khaleque MA, Sawyer DB, Ciocca DR (2006) Heat shock proteins in cancer: chaperones of tumorigenesis. Trends Biochem Sci 31: 164–172. 16483782 10.1016/j.tibs.2006.01.006

[11] Gupta RS, Ramachandra NB, Bowes T, Singh B (2008) Unusual cellular disposition of the mitochondrial molecular chaperones Hsp60, Hsp70 and Hsp10. Novartis Found Symp 291: 59–68; discussion 69–73, 137–140. 10.1002/9780470754030.ch5 18575266

[12] Myung JK, Afjehi-Sadat L, Felizardo-Cabatic M, Slavc I, Lubec G (2004) Expressional patterns of chaperones in ten human tumor cell lines. Proteome Sci 2: 8. 15598346 10.1186/1477-5956-2-8 PMC543454

[13] Pace A, Barone G, Lauria A, Martorana A, Piccionello AP, et al (2013) Hsp60, a novel target for antitumor therapy: structure-function features and prospective drugs design. Curr Pharm Des 19: 2757–2764. 23092316 10.2174/1381612811319150011

[14] Faried A, Sohda M, Nakajima M, Miyazaki T, Kato H, et al (2004) Expression of heat-shock protein Hsp60 correlated with the apoptotic index and patient prognosis in human oesophageal squamous cell carcinoma. Eur J Cancer 40: 2804–2811. 15571964 10.1016/j.ejca.2004.08.013

[15] Cappello F, Di Stefano A, D’Anna SE, Donner CF, Zummo G (2005) Immunopositivity of heat shock protein 60 as a biomarker of bronchial carcinogenesis. Lancet Oncol 6: 816. 16198989 10.1016/S1470-2045(05)70393-4

[16] Hamelin C, Cornut E, Poirier F, Pons S, Beaulieu C, et al (2011) Identification and verification of heat shock protein 60 as a potential serum marker for colorectal cancer. FEBS J 278: 4845–4859. 21973086 10.1111/j.1742-4658.2011.08385.x PMC3265716

[17] Khalil AA (2007) Biomarker discovery: a proteomic approach for brain cancer profiling. Cancer Sci 98: 201–213. 17233837 10.1111/j.1349-7006.2007.00374.x PMC11158801

[18] Chu D, Zhang Z, Li Y, Zheng J, Dong G, et al (2011) Matrix metalloproteinase-9 is associated with disease-free survival and overall survival in patients with gastric cancer. Int J Cancer 129: 887–895. 20957628 10.1002/ijc.25734

[19] Edge SB, Compton CC (2010) The American Joint Committee on Cancer: the 7th edition of the AJCC cancer staging manual and the future of TNM. Ann Surg Oncol 17: 1471–1474. 20180029 10.1245/s10434-010-0985-4

[20] Wang J, Cui S, Zhang X, Wu Y, Tang H (2013) High expression of heat shock protein 90 is associated with tumor aggressiveness and poor prognosis in patients with advanced gastric cancer. PLoS One 8: e62876. 23638161 10.1371/journal.pone.0062876 PMC3637377

[21] Tanaka A, Kamada T, Yokota K, Shiotani A, Hata J, et al (2009) Helicobacter pylori heat shock protein 60 antibodies are associated with gastric cancer. Pathol Res Pract 205: 690–694. 19450936 10.1016/j.prp.2009.04.008

[22] Cappello F, David S, Rappa F, Bucchieri F, Marasa L, et al (2005) The expression of HSP60 and HSP10 in large bowel carcinomas with lymph node metastase. BMC Cancer 5: 139. 16253146 10.1186/1471-2407-5-139 PMC1289279

[23] Cappello F, Di Stefano A, David S, Rappa F, Anzalone R, et al (2006) Hsp60 and Hsp10 down-regulation predicts bronchial epithelial carcinogenesis in smokers with chronic obstructive pulmonary disease. Cancer 107: 2417–2424. 17048249 10.1002/cncr.22265

[24] Nelson AR, Fingleton B, Rothenberg ML, Matrisian LM (2000) Matrix metalloproteinases: biologic activity and clinical implications. J Clin Oncol 18: 1135–1149. 10694567 10.1200/JCO.2000.18.5.1135

[25] Zhao F, Zhang Q, Kang C, Cui X, Wang T, et al (2010) Suppression of matrix metalloproteinase-9 expression by RNA interference inhibits SGC7901 gastric adenocarcinoma cell growth and invasion in vitro and in vivo. Med Oncol 27: 774–784. 19680827 10.1007/s12032-009-9285-x

[26] Lin CS, He PJ, Tsai NM, Li CH, Yang SC, et al (2010) A potential role for Helicobacter pylori heat shock protein 60 in gastric tumorigenesis. Biochem Biophys Res Commun 392: 183–189. 20060384 10.1016/j.bbrc.2010.01.010

[27] Cappello F, Marino Gammazza A, Palumbo Piccionello A, Campanella C, Pace A, et al (2014) Hsp60 chaperonopathies and chaperonotherapy: targets and agents. Expert Opin Ther Targets 18: 185–208. 24286280 10.1517/14728222.2014.856417

